# Microbial community analysis in biocathode microbial fuel cells packed with different materials

**DOI:** 10.1186/2191-0855-2-21

**Published:** 2012-03-29

**Authors:** Yanmei Sun, Jincheng Wei, Peng Liang, Xia Huang

**Affiliations:** 1 State Key Joint Laboratory of Environment Simulation and Pollution Control, School of Environment, Tsinghua University, Beijing 100084, P. R. China

**Keywords:** Biocathode microbial fuel cell, Cathodic materials, Electricity generation, Microbial community

## Abstract

Biocathode MFCs using microorganisms as catalysts have important advantages in lowering cost and improving sustainability. Electrode materials and microbial synergy determines biocathode MFCs performance. In this study, four materials, granular activated carbon (GAC), granular semicoke (GS), granular graphite (GG) and carbon felt cube (CFC) were used as packed cathodic materials. The microbial composition on each material and its correlation with the electricity generation performance of MFCs were investigated. Results showed that different biocathode materials had an important effect on the type of microbial species in biocathode MFCs. The microbes belonging to Bacteroidetes and Proteobacteria were the dominant phyla in the four materials packed biocathode MFCs. *Comamonas *of Betaproteobacteria might play significant roles in electron transfer process of GAC, GS and CFC packed biocathode MFCs, while in GG packed MFC *Acidovorax *may be correlated with power generation. The biocathode materials also had influence on the microbial diversity and evenness, but the differences in them were not positively related to the power production.

## Introduction

Microbial fuel cells (MFCs) utilize microorganisms as catalysts, which can promote biodegradation of organic matters and simultaneously produce an electrical current (Bond et al. 2002). In the past few years, researchers generally use chemical cathode MFC to remove the organic carbon in wastewater, but the cost of chemical cathode is high and it is easily lead to pollution. Currently, biocathode MFCs using microorganisms instead of common Pt as catalysts have important advantages in lowering cost, expanding function and improving sustainability. Therefore, biocathode MFCs as a new economical and environmentally friendly wastewater treatment technology has drawn more and more attentions (Huang et al. 2011). Although biocathode MFCs have many advantages, the current studies are still at laboratory level. The main challenge for their large-scale application is low power generation capability. Microorganisms are the core of biocathode MFCs. In the anode, microorganisms attaching on the electrode material and forming biofilm play an essential role in MFC generating electricity (Rabaey and Rozendal 2010), and in the cathode, the microbial catalytic efficiency plays a key role to improve the cathode potential and power output (Osman et al. 2010). Therefore, better understanding of the ecology of the microbial communities in the different reactors will be helpful to improve MFCs power production.

At present, the anodic microbes get more attention, including the electricity-producing bacteria species ([Bibr B11][Bibr B27]), anodic microbial community composition ([Bibr B3][Bibr B13][Bibr B16][Bibr B28]), the mechanism of extracellular electron transfer ([Bibr B4][Bibr B24]) and so on. In contrast, the researches on the microbes of biocathode MFCs are very limited, and mainly focused on the role of pure bacteria in biocathode MFCs. For instance, Carbajosa et al. (2010) found that an acidophilic *Acidithiobacillus ferrooxidans *could promote oxygen reduction in biocathode MFCs. Mao et al. (2010) reported that the power generation from a biocathode MFC was biocatalyzed by ferro/manganese-oxidizing bacteria. Recently, a research analyzed the microbial community and electron transfer, when nitrate was used as electron acceptor (Chen et al. 2010). However, electrode materials and microbial synergy determines biocathode MFCs performance. Different electrode materials have certain differences in conductivity, surface area and porosity. These differences may affect the cathode microbial adhesion and growth. However, the influence of different biocathode materials on the microbial composition is still unknown.

In our previous study (Wei et al. 2011), two types of relative cheaper electrode materials, granular semicoke (GS) and granular activated carbon (GAC), as biocathode packed materials, and the material characteristic, electrochemical performance and price-performance ratio were compared with carbon felt cube (CFC) and granular graphite (GG). Results indicated that MFCs with GS and GAC outperformed MFCs with GG and CFC biocathode. But the dominate microorganisms in different biocathode materials were not analyzed and the interaction mechanism between microbes and biocathode materials was unclear.

The objective of this study is to analyze the microbial community composition attaching on the four biocathode materials, illustrate the predominate microbes on each biocathode materials and analyze the relationship between microorganisms and power generation in biocathode MFCs.

## Materials and methods

### MFC construction and operation

Four double-chambered flat plate MFCs with same size were built. Each MFC had two compartments with a total volume of 100 mL (2 cm thickness, 50 cm^2 ^cross section), which were separated by an Ultrex cation exchange membrane (CMI-7000, Membranes International, USA). The titanium mesh was placed next to the cation exchange membrane, which was used to gather electrons flowing in each chamber. The titanium sheet was served as a lead to connect both electrodes and external resistance. Four biocathode materials (CFC, GG, GAC and GS) were filled in separate cathodic compartments, and anodic compartments of all four MFCs were filled with the same CFC used in cathode. The anodic and cathodic compartments were inoculated with microbial consortiums previously enriched in biocathode MFCs that had been operated in fed-batch mode for over three months. Two saturated calomel electrodes (SCE, 0.242 V vs. standard hydrogen electrode (SHE), Leici, China) were fitted through the rubber stopper of anodic and cathodic compartments respectively to be used as reference electrodes. The medium in the anodic compartment consisted of 1.64 g/L CH_3_COONa, 1.5 g/L NH_4_Cl, 3.4 g/L KH_2_PO_4_, 4.4 g/L KH_2_PO_4_, 0.1 g/L CaCl_2_·2H_2_O and 0.1 g/L MgCl_2_·6H_2_O. The cathode was fed with a similar medium with the anode, except that 1.64 g/L CH_3_COONa was replaced with 1.90 g/L NaHCO_3_. The nutrient solution was pumped to anodic and cathodic compartments separately at a rate of 20 mL/min using a peristaltic pumps (BT100-1 L, Baoding Longer Precision Pump Co., Ltd., China). To provide abundant oxygen for the cathodic reaction, air was continuously sparged into a conical flask which was connected with the vessel of cathode recirculation. MFCs were operated in fed-batch mode, and the anolyte and catholyte were replaced every 3 days. All reactors were operated at ambient temperature (28 ± 3°C) with a 1000 Ω resistor connected unless otherwise specified.

### Material characteristics and electrochemical analysis

The specific surface area of four cathodic materials was measured with a micropore surface area and pore size analyzer (Autosorb-1 MP, Quantachrome Instrumants, USA).

The voltage across an external resistor was measured at a time interval of 20 min using a data acquisition system (DAQ2213, ADLINK, Beijing, China). The power densities were calculated from *P *= *IE*/*V*, where *I *is the current (=*E*/*R*), *E *the measured voltage, *R *is the external resistance, *P *the power density, and *V *the net liquid volume of the anodic compartment (Logan et al., 2006).

### Bacterial community analysis

Biofilms attaching on the biocathode materials in MFCs were sampled at the end of the experiment, and rinsed with sterile distilled water to remove loosely attached bacteria. The cathodic biofilm DNA was extracted using a PowerSoil DNA isolation kit (MO BIO Laboratories) and then the bacteria 16S rRNA was amplified. Bacterial universal primers 27f (5'-AGA GTT TGA TCM TGG CTC AG-3') and 1495r (5'-CTA CGG CTA CCT TGT TAC GA-3') (Barberio et al. 2001) were used, and the PCR conditions were carried out in accordance with the reference (Di Cello et al. 1997). The products of triplicate PCR amplifications from each sample were joined and purified with Wizard^® ^SV Gel and PCR Clean-Up System (Promega). The purified product was ligated into the pGEM^®^-T Easy vector (Promega) and the resulting plasmids were used to transform competent *E. coli *JM109 cells. Transformants were screened using blue-white selection on Luria agar containing

X-gal/IPTG and 100 mg/mL ampicillin. White colonies were selected, transferred to fresh ampicillin-supplemented plates and incubated overnight at 37°C. Plasmids were extracted using EZ Spin Column Plasmid Mini-Preps Kit (Shanghai Sangon). Positive clones were sequenced with primers T7/SP6 by a DNA sequencer (ABI 3730). Clone sequences were identified by comparison to the Genbank nucleotide database using BLAST via the National Center for Biotechnology Information (NCBI) (http://www.ncbi.nlm.nih.gov/BLAST/). Mega 4.0.2 (Tamura et al. 2007) was used to align these sequences, and the neighbor-joining method was employed to generate a phylogenetic tree with a bootstrap test (1000 replicates) of phylogeny. Sequences obtained in this study have been deposited in GenBank with accession numbers of JN541127-JN541192 and JN565978. Sequences with greater than 97% identity are typically assigned to the same species (Schloss and Handelsman 2005). Rarefaction curves were used to determine if a sufficient number of clones from each of the libraries had been sequenced (http://strata.uga.edu/software/Software.html). The sampling coverage value, diversity (Shannon-Wiener index) and evenness were calculated as previously described (Gremion et al. 2003).

## Results

### MFC performance

The maximum power densities of four different cathodic materials packed MFCs are shown in Additional file 1: Table S1. Results indicated that as cathodic material, the power density of GAC was the highest (24.3 W/m^3^), followed by GS (20.1 W/m^3^), CFC (17.1 W/m^3^), and GG (14.4 W/m^3^).

### Diversity estimation and clone library comparison

Four 16S rRNA gene libraries were constructed to analyze the effect of biocathode materials on microbial composition. About 60 positive clones were sequenced in each library. Rarefaction curves (Additional file 1: Figure S1) showed that the number of clones was high enough to cover almost all the OTUs (operational taxonomic units) in samples. The coverage value for each clone library was more than 80%, which also indicated that the four clone libraries could reflect the microbial community composition in samples. There were 22, 21, 16 and 13 genotypes in GS, CFC, GG and GAC clone libraries, respectively (Table 1). In GS packed MFCs, microbial diversity was the highest, the Shannon-Weaver index was 2.70, followed by the GG and CFC packed MFCs, and GAC packed MFC had the lowest microbial diversity, about 1.83. Species evenness distributed on each electrode material had the similar trend to diversity. In GS packed MFC, the distribution of species was relatively average compared with other packed materials (Table 1).

**Table 1:** Bacterial 16S rRNA clone libraries from different cathode material packed in MFCs

Material	Transformant number	Positive clones number	OTUs	Coverage (%)	Shannon index (*H'*)	Evenness (E)
GS	70	63	22	84.62	2.70	0.63

GG	68	67	21	80.60	2.49	0.53

CFC	59	55	16	83.64	2.25	0.51

GAC	72	68	13	89.71	1.83	0.41

OTUs: operational taxonomic units

### Microbial community distribution

**Figure 1:**
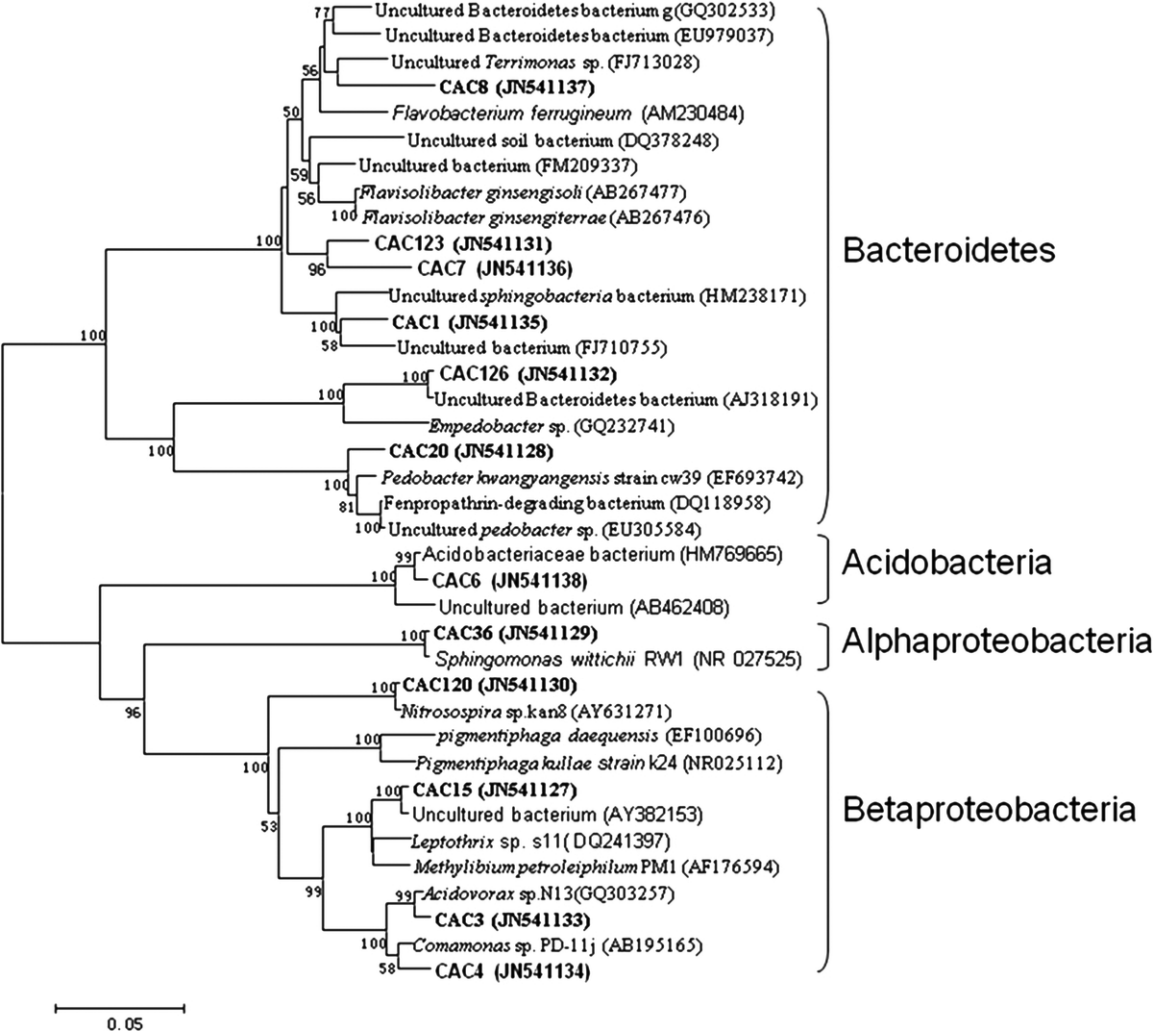
**Neighbor-joining tree showing the phylogenetic relationship of bacteria 16S rRNA acquired from the activated carbon packed MFC**. The clones with labels CAC are from cathodic activated carbon. Bootstrap values are only shown for nodes that had > 50% support in bootstrap analysis of 1000 replicates in trees. The scale bar indicates 5% estimated sequence divergence.

**Figure 2:**
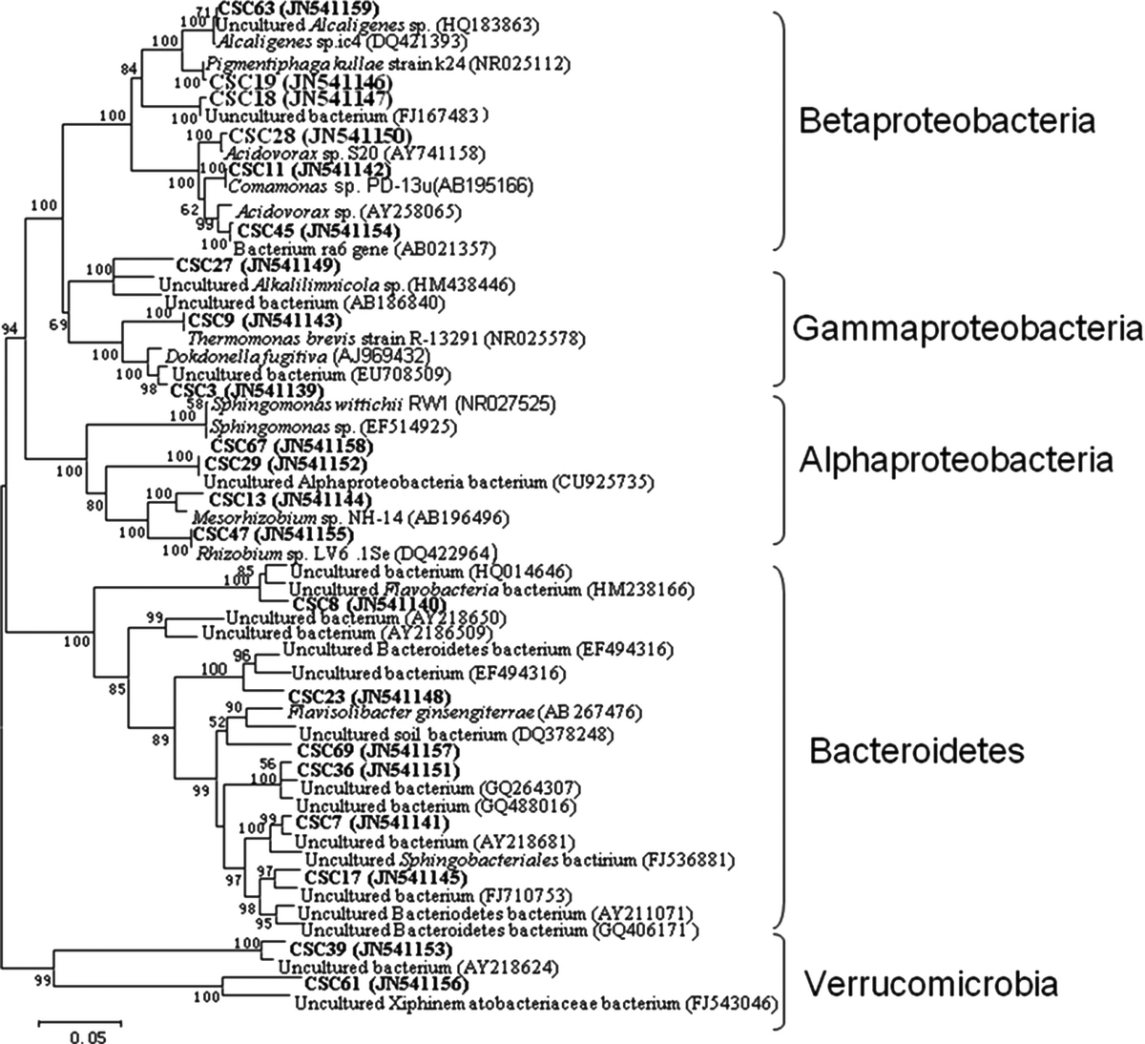
**Neighbor-joining tree showing the phylogenetic relationship of bacteria 16S rRNA acquired from the semicoke packed MFC**. The clones with labels CSC are from cathodic semicoke. Bootstrap values are only shown for nodes that had > 50% support in bootstrap analysis of 1000 replicates in trees. The scale bar indicates 5% estimated sequence divergence.

**Figure 3:**
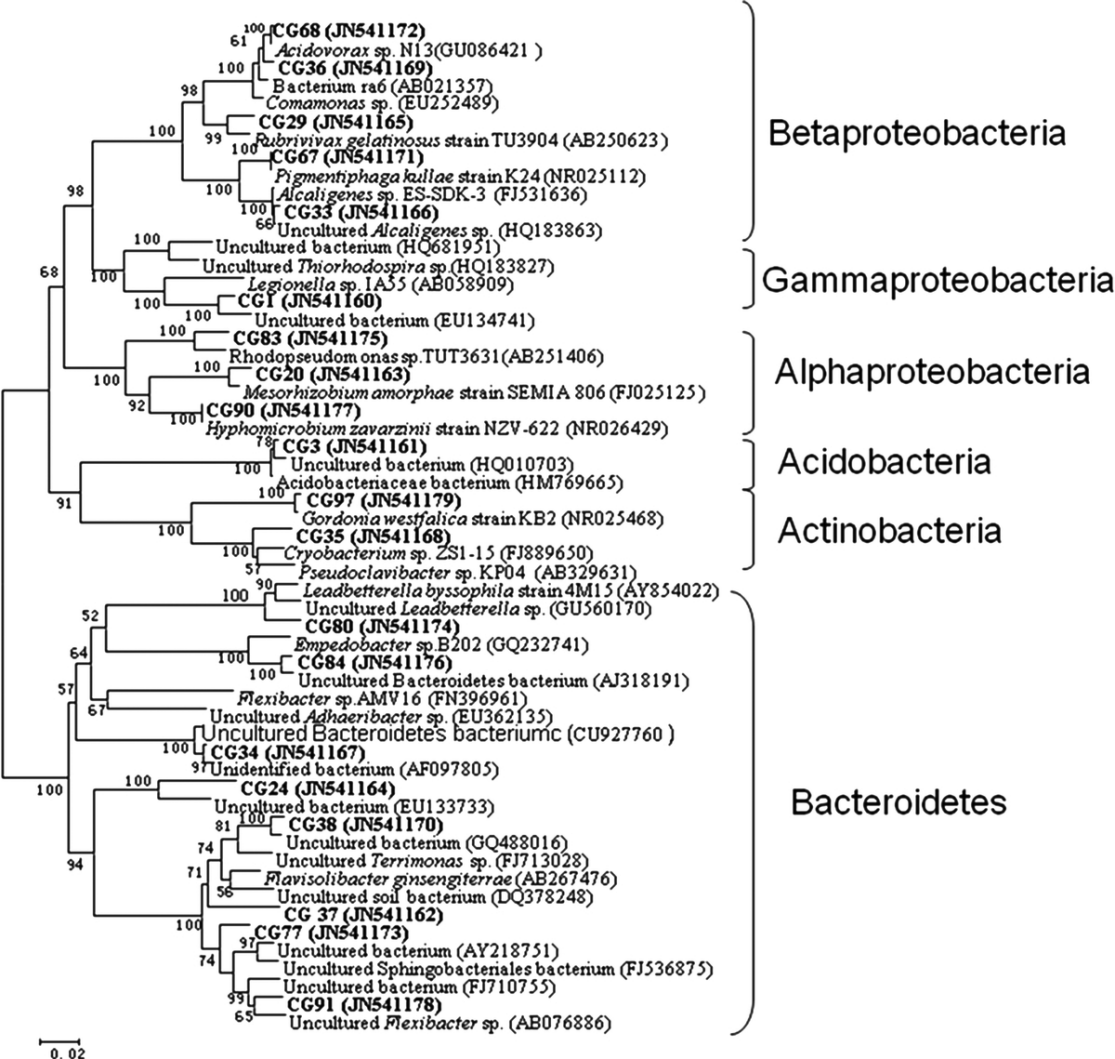
**Neighbor-joining tree showing the phylogenetic relationship of bacteria 16S rRNA acquired from graphite packed MFC**. The clones with labels CG are from cathodic graphite. Bootstrap values are only shown for nodes that had > 50% support in bootstrap analysis of 1000 replicates in trees. The scale bar indicates 2% estimated sequence divergence.

**Figure 4:**
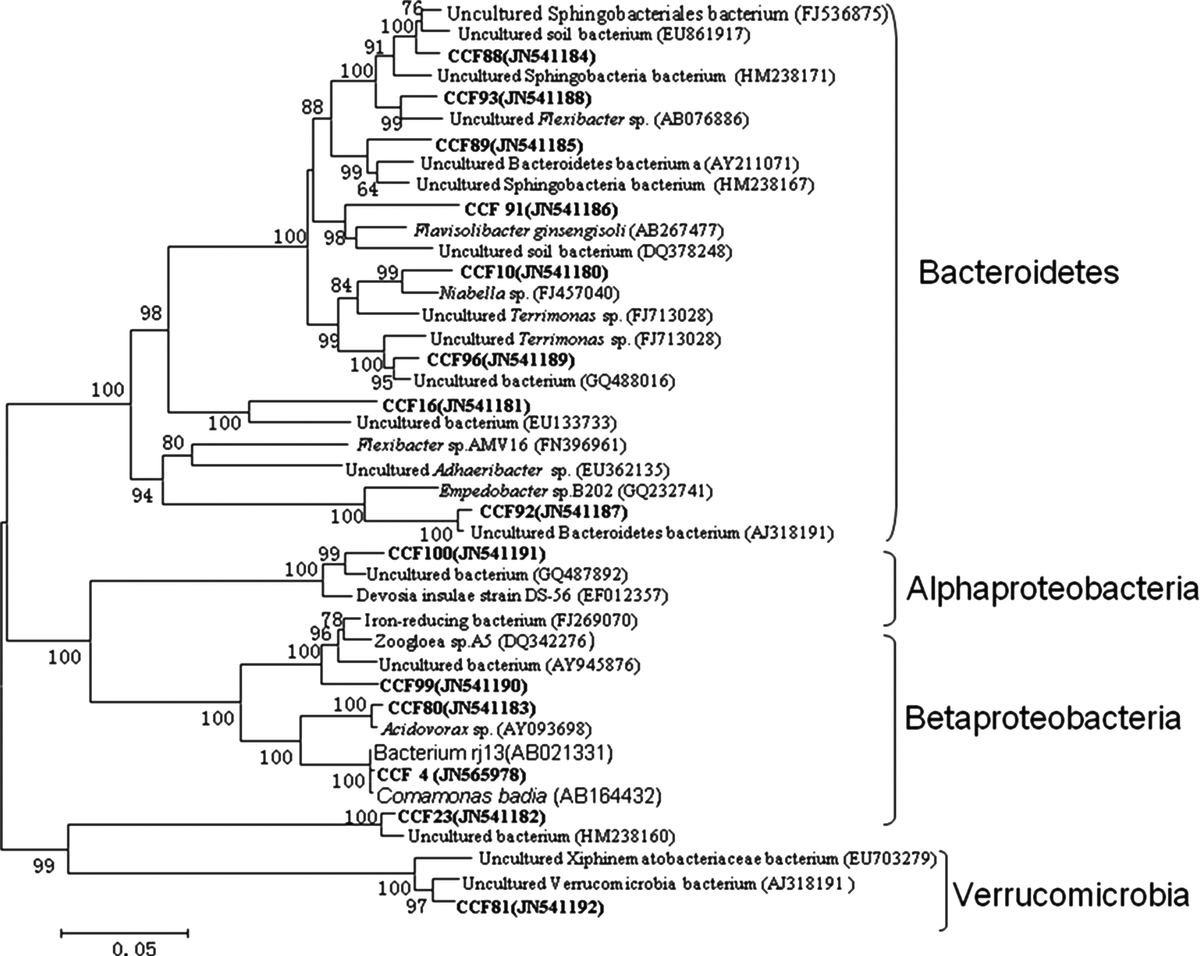
**Neighbor-joining tree showing the phylogenetic relationship of bacteria 16S rRNA acquired from carbon felt packed MFC**. The clones with labels CCF are from cathodic carbon felt. Bootstrap values are only shown for nodes that had > 50% support in bootstrap analysis of 1000 replicates in trees. The scale bar indicates 5% estimated sequence divergence.

The microbial community distribution on each biocathode material was compared and four phylogenetic trees were constructed to characterize the relationship between those clone sequences. Results showed that microorganisms in the GAC packed MFC could be assigned into four groups, consisting of Alphaproteobacteria, Betaproteobacteria, Bacteroidetes and Acidobacteria (Figure 1). In the GS packed MFC, the cathodic biofilm was dominated by Alphaproteobacteria, Betaproteobacteria, Gammaproteobacteria, Bacteroidetes and Verrucomicrobia (Figure 2). The microorganisms belonged to Alphaproteobacteria, Betaproteobacteria, Gammaproteobacteria. Bacteroidetes also existed in GG packed MFC. In addition to the bacterial phyla, Acidobacteria and Actinobacteria were also found in GG (Figure 3). Alphaproteobacteria Betaproteobacteria, Bacteroidetes and Verrucomicrobia were dominant phyla attached to CFC (Figure 4).

**Figure 5:**
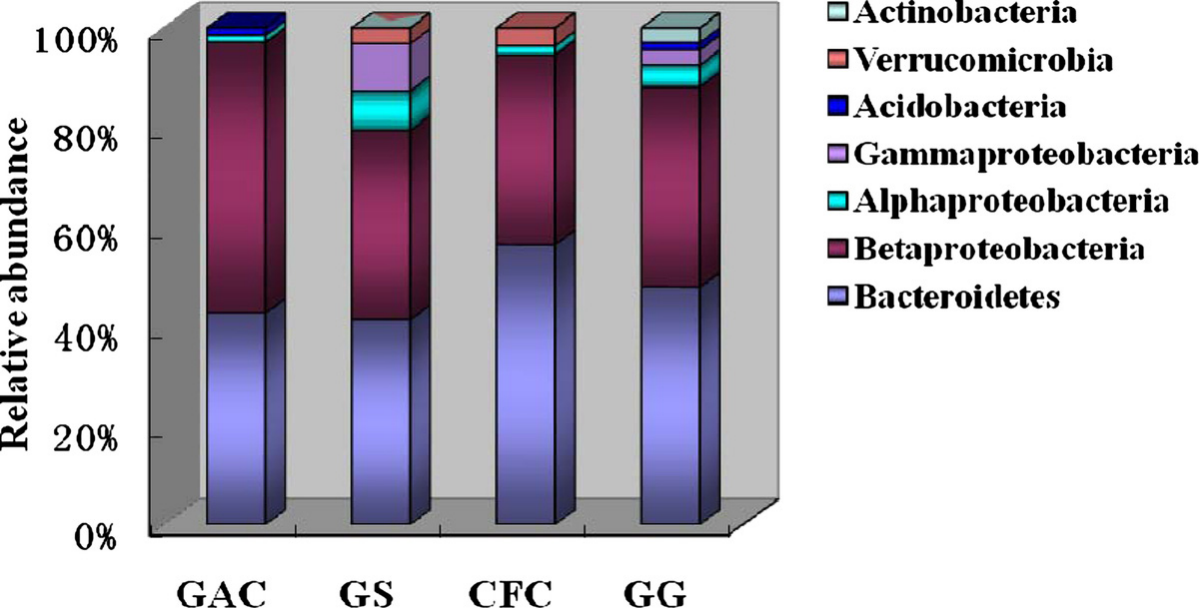
**Microbial community distribution and relative abundance in different biocathode materials packed MFCs**. GAC (granular activated carbon), CFC (carbon felt cube), GG (granular graphite), GS (granular semicoke).

The relative abundance of each phylum on the four biocathode materials was analyzed and compared in Figure 5. Results suggested that Bacteroidetes and Betaproteobacteria were the most abundant in the biofilms sampled from four cathode materials (Figure 5). The two phyla accounted for more than 80% of the total population in each biocathode material. The relative abundances of Alphaproteobacteria and Gammaproteobacteria were higher in GS compared with other materials. Actinobacteria was only existed in the GG cathodic material.

**Figure 6:**
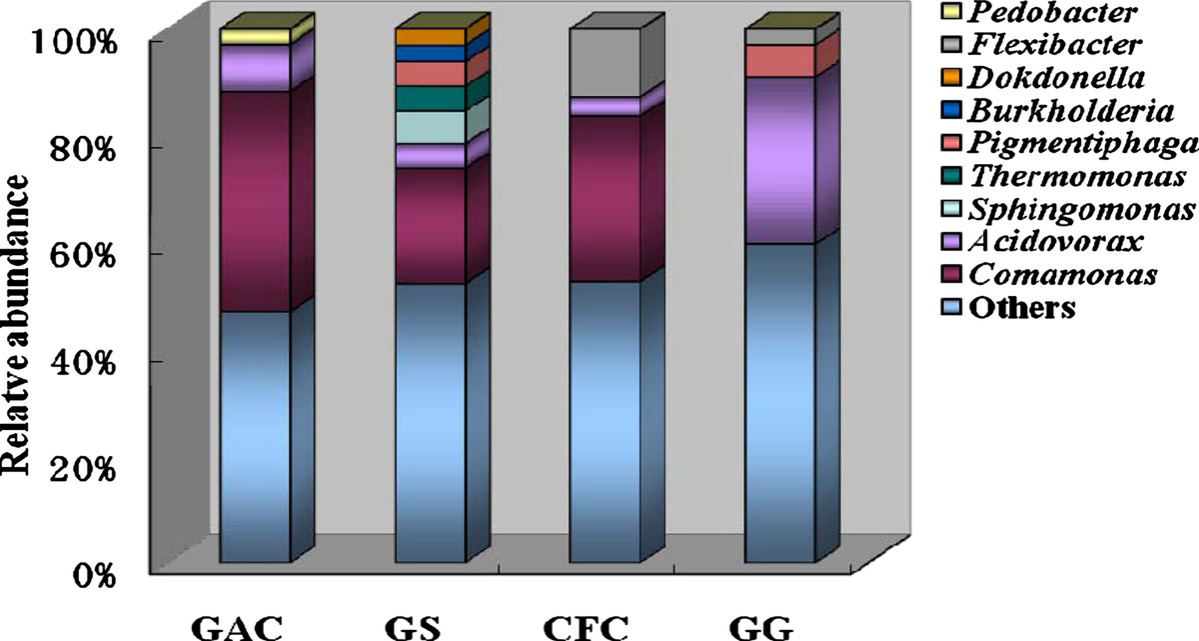
**The dominant genus and relative abundance in different materials packed biocathode MFCs**. Others: the collection of microbial abundance less than 2% and the undefined microbes. GAC (granular activated carbon), CFC (carbon felt cube), GG (granular graphite), GS (granular semicoke).

To visualize specific species and relative abundance within each electrode material, column chart was performed to illustrate the microbial community composition, as shown in Figure 6. The dominate genus and relative abundance varied significantly among the four cathodic materials. In the GAC packed MFC, clones showed a great similarity to *Comamonas*, and the amount of *Comamonas *accounted for 41% of the population. In addition, *Acidovorax *attaching on the GAC packed MFC, consisted of 9% of the library. *Pedobacter *can only be found in GAC, and the ratio of its population to the library was 3%. The abundance of *Comamonas *was also high in the GS packed MFC, with a percentage of 21%. *Acidovorax *and *Pigmentiphaga *can also be found in GS, and each genus took up approximately 5% of the microbial community. *Sphingomonas*, *Thermomonas*, *Burkholderia *and *Dokdonella *can only be retrieved from cathodic communities of the CFC packed MFC, and they covered 6%, 5%, 3% and 3% of the library, respectively. In the case of CFC, the dominate group was similar to GAC and GS, which was also dominated by *Comamonas *and comprised 30% of the population. The relative abundance of *Flexibacter *was high in CFC, and it accounted for 13% of the library. Different from GAC, GS and CFC packed MFCs, the biofilm on GG was dominated by *Acidovorax*, which formed 31% of the community. The microbes belonging to *Pigmentiphaga *and *Flexibacter *can also be detected on GG.

## Discussion

The maximum power densities of GAC, GS,CFC and GG packed MFCs showed a decreasing trend. The specific areas changed in the similar way as the power densities of MFCs with different cathodic materials. It indicated that the power density had positive correlation with specific area, which can be assured that high specific area is profitable for microbial attachment and biological catalytic processes.

Shannon diversity indices results suggested that the diversity was the highest on GS and lowest on GAC. The results showed that biocathode materials had effects on the microbial diversity and evenness. Results on electrical power showed that the power density was the highest in GAC packed MFC, followed by GS, CFC and GG (Additional file 1: Table S1). It indicated that the microbial diversity and evenness in biocathode material was not positively related to the power production. Other studies about anodic microbes also showed that numerical abundance of microorganisms in anodic biofilms was not correlated with the current (Kiely et al. 2010).

Phylogenetic analysis indicated that the biofilm developing on each cathodic surface had some differences. It could be assumed that the microbial members had different affinities for the materials. Bacteroidetes and Betaproteobacteria were predominant in the biofilms sampled from four cathode materials. In a related study, it was also reported that the microbial community composition of an oxygen reducing biocathode was dominated by Bacteroidetes (Rabaey et al. 2008). When nitrate was served as the terminal electron acceptor on the biocathode, Chen et al. (2010) analyzed the microbial community dynamics and the results showed that Betaproteobacteria and Bacteroidetes were the most abundant division of the community. Another study showed that Gammaproteobacteria was the most abundant, followed by uncultured Bacteria and Bacteroidetes (Chen et al. 2008). These results indicated that the microorganisms belonging to proteobacteria and Bacteroidetes play important roles in catalyzing oxygen or nitrate reduction in cathodic compartment.

Electrode materials had an important effect on the type of microbial species in MFCs reactors. Different electrode materials have different microscopic surface structure and conductivity, which in turn affects the adhesion of specific microbes ([Bibr B20][Bibr B29]). In GAC, GS and CFC packed MFCs, *Comamonas *of Betaproteobacteria was the dominated genus; *Acidovorax *was the most obvious microbes of GG packed MFC. The powder density was the highest in GAC packed MFC, followed by GS, CFC and GG. Therefore microbes *Comamonas *and its abundance in cathodic materials might have some positive relation to the power generation in GAC, GS and GG packed MFCs, while in GG packed MFC *Acidovorax *may be correlated with electron transfer. A recent study showed that *Comamonas testosteroni *displayed a higher power production performance under a high pH condition in the anode chamber (Juang et al. 2011). The similarities between anode and cathode reducing/oxdizing populations may indicate the capability of many organisms to perform electron transfer both to and from electrodes, such as *Shewanella putrefaciens *and *Geobacter sulfurreducens*. Some electrochemically active bacteria in biocathodes have been reported, including Gram-negative and positive bacteria, such as *Acinetobacter calcoaceticus*, *Sphingobacterium multivorum*, *Micrococcus luteus *and *Bacillus subtilis*, which can catalyze oxygen reduction in biocathode MFCs ([Bibr B21][Bibr B8]). Therefore, the cathodic biofilm in a MFC is composed of diverse populations of bacteria, and they may work together to electron transfer and power production.

In conclusion, electrical results showed that the power density was the highest in GAC packed MFC, followed by GS, CFC and GG. Different biocathode materials had effects on the microbial diversity and evenness, but the differences in microbial diversity and evenness of different biocathode materials were not positively related to the power production. Biocathode materials had an important effect on the type of microbial species in MFCs reactors. The microbes belonging to Bacteroidetes and Proteobacteria were the dominant phyla in the four materials packed biocathode MFCs. *Comamonas *of Betaproteobacteria might have important effects on electron transfer process of GAC, GS and CFC packed biocathode MFCs, while in GG packed MFC *Acidovorax *may be correlated with power generation.

## Competing interests

The authors declare that they have no competing interests.

## Supplementary Material

Additional file 1 **Figure S1**. Rarefaction analysis of four clone libraries including GAC (granular activated carbon), CFC (carbon felt cube), GG (granular graphite) and GS (granular semicoke). **Table S1**. The specific area and power density of different cathodic materials packed MFCs (Wei et al. 2011). Click here for file
