# Supplementary material for: Microbial community analysis in biocathode microbial fuel cells packed with different materials
Source: AMB Express. 2012 Mar 29;2:21. doi: 10.1186/2191-0855-2-21 (PMC3349616; doi:10.1186/2191-0855-2-21)
Supplement: Additional file 1 — Figure S1. Rarefaction analysis of four clone libraries including GAC (granular activated carbon), CFC (carbon felt cube), GG (granular graphite) and GS (granular semicoke). Table S1. The specific area and power density of different cathodic materials packed MFCs (Wei et al. 2011). [file 2191-0855-2-21-S1.DOC]

*Supporting Information for manuscript*

**Microbial community analysis in** **biocathode microbial fuel cells packed with different materials**

**Yanmei Sun, Jincheng Wei, Peng Liang, Xia Huang***

*State Key Joint Laboratory of Environment Simulation and Pollution Control*

*School of Environment, Tsinghua University, Beijing, 100084, P.R. China*

**Corresponding author: Tel: +86 10 62772324; Fax: +86 10 62771472;*

*E-mail: xhuang@tsinghua.edu.cn*


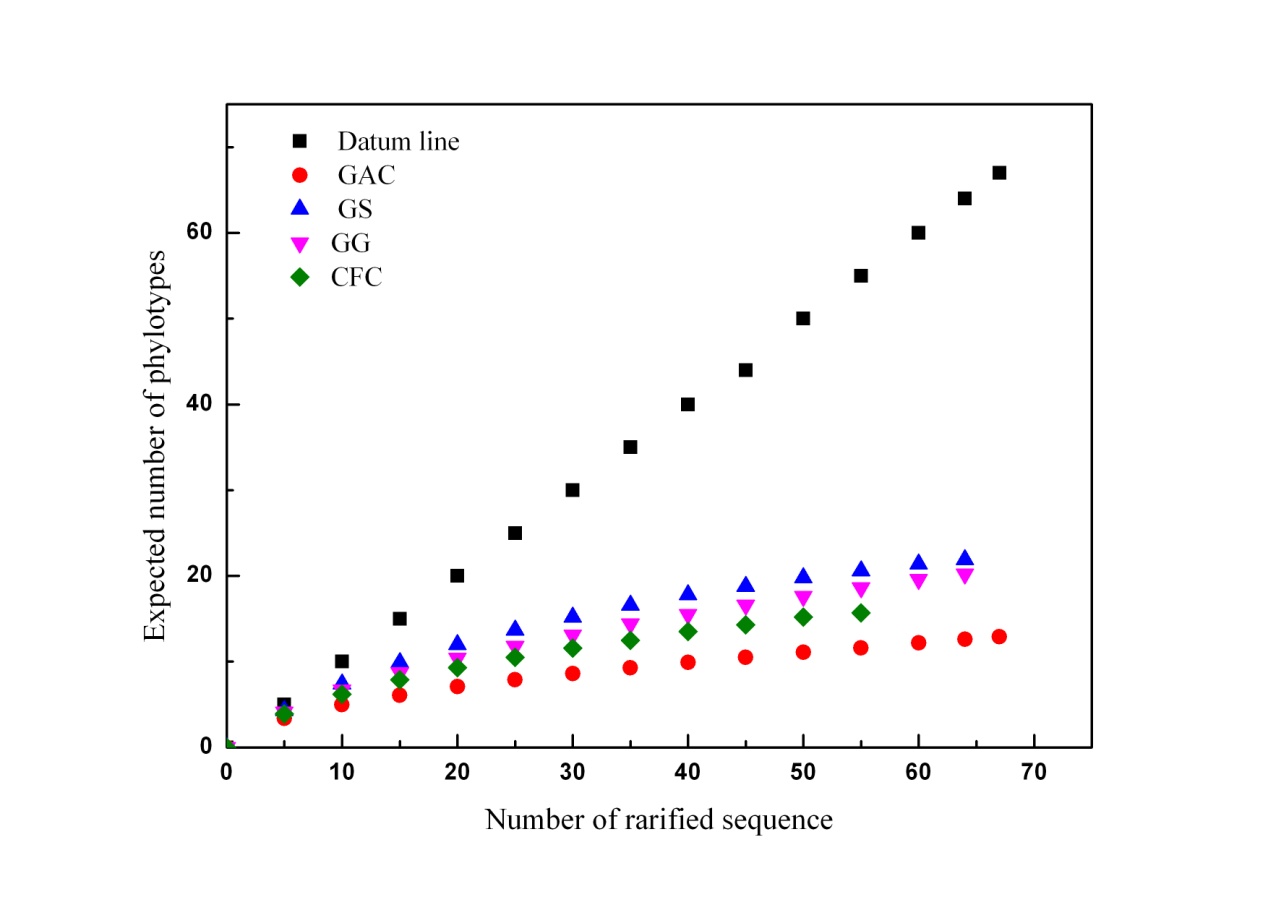


**Fig. S1** Rarefaction analysis of four clone libraries including [GAC](http://dict.cnki.net/dict_result.aspx?searchword=颗粒状活性炭&tjType=sentence&style=&t=granule+activated+carbon) ([granular activated carbon](http://dict.cnki.net/dict_result.aspx?searchword=颗粒状活性炭&tjType=sentence&style=&t=granule+activated+carbon)), CFC (carbon felt cube), GG (granular graphite) and GS (granular semicoke).

**Table S1** The specific area and power density of different cathodic materials packed MFCs (Wei et al. 2011).

| Material | Specific area (g/m^2^) | Maximum power density (W/m^3^) |
| --- | --- | --- |
| GAC | 686 | 24.3 |
| GS | 236 | 20.1 |
| CFC | 0.845 | 17.1 |
| GG | 0.623 | 14.1 |
